# The Meaning of Plants' Names: A New Discovering Approach to Its Medicinal and/or Toxic Properties

**DOI:** 10.1155/2024/6678557

**Published:** 2024-02-19

**Authors:** Letícia dos Santos Dantas Lima, Luiz Felipe Domingues Passero, Alexandre Indriunas, Ingrid de Souza Santos, Luíza Francisco Uchôa Coqueiro, Kayo Alexandre Souza da Cruz, Adriana Batista de Almeida, José Carlos Fernandes Galduróz, Eliana Rodrigues

**Affiliations:** ^1^Center for Ethnobotanical and Ethnopharmacological Studies (CEE), Department of Environmental Sciences, Universidade Federal de Sa˜o Paulo (UNIFESP), Rua Prof. Artur Riedel, No 275, Diadema 09972-270, SP, Brazil; ^2^São Paulo State University (UNESP), Institute of Biosciences, Praça Infante Dom Henrique, s/n, São Vicente 11330-900, SP, Brazil; ^3^Institute for Advanced Studies of Ocean, São Paulo State University (UNESP), São Vicente 11350-011, Brazil; ^4^Museu Nacional, Universidade Federal do Rio de Janeiro, Quinta da Boa Vista-São Cristóvão, Rio de Janeiro 20940-040, Brazil; ^5^Departament of Psychobiology, Universidade Federal de São Paulo, Rua Botucatu, 862, Edifício Ciências Biomédicas-1° Andar, São Paulo 04724-000, Brazil

## Abstract

Some of the vernacular or scientific names are related to possible medicinal and/or toxic properties that can reveal the presence of potential bioactive agents, contributing to the discovery of new drugs and/or knowledge of the risks associated with their use. This study sought to list the scientific and vernacular names of plants whose lexicons are related to those possible properties of plants and to compare them with the “ethno” (ethnobotanical and ethnopharmacological) and pharmacological data available in the scientific literature. A floating reading of the two classical and reference works on Brazilian medicinal plants was performed, and plants with vernacular and/or scientific names related to the possible medicinal and/or toxic properties were listed. Correlations between the meanings of the species' names (lexicon) and their possible biological properties were made from their translation from Latin by consulting dictionaries. A bibliographic survey was conducted on the “ethno” and pharmacological data for each species. Finally, data from these three dimensions (lexicon, “ethno,” and pharmacology) were classified and compared using a bioprospection classification. It resulted in a list of 90 plant species belonging to 47 families. 66 of the 90 species presented “ethno” data from the scientific literature, while 46 species presented pharmacological data. Of these, 46 (69.7%) and 27 (58.7%), respectively, showed equivalence with the possible medicinal and/or toxic properties of plants according to their lexicons. According to this study, half of the plants investigated demonstrate equivalence in the three dimensions analyzed (lexicons, “ethno,” and pharmacological data from the scientific literature). Gastrointestinal and nervous system categories are among the most common in all three dimensions. Plant lexicons may be closely linked to the possible medicinal and/or toxic properties and the study of plant lexicons may represent one more approach for the search for new drugs, mainly considering the gastrointestinal, nervous, and parasites categories.

## 1. Introduction

Taxonomy, which is based on identification, description, nomenclature, and classification, is an extremely important area and one of the oldest disciplines in biology [1, 2]. A taxon, a grouping of defined organisms in which living beings are classified hierarchically [3], follows a classification code consolidated by Carolus Linnaeus in the 18th century. This classification is based on a binomial system to define the genus and species of each living organism. Currently, the botanical nomenclature for algae, fungi, and plants is regulated by the International Nomenclature Code (CIN), but it still follows the binomial standard proposed by Linnaeus. The binomial is composed of the genus, specific epithet, and followed by the name(s) of the author(s). Both generic names and epithets are always latinized and follow the international code. It is important to note that although each taxonomic group has only one valid name, a group can present several synonyms according to its classification over time [4]. In addition, consensus among taxonomists about a valid name is not always reached on the accuracy of the plant nomenclature. Thus, there are ongoing debates on the validity of a given botanical name.

In general, the generic epithet provides information on the taxon to which the organism belongs, and the specific epithet can refer to the morphological, environmental, or toponymic characteristics of the plant, to popular knowledge of the plant, and to its organoleptic properties, biological activities, medicinal/toxic applications, or the honour of people and others [5, 6]. In this sense, an interesting example is *Papaver somniferum*, whose generic epithet refers to the family to which it belongs “Papaveraceae,” and the specific epithet “somniferum” is associated with sleep, which in turn is correlated with the pharmacological activity of opium and morphine, which are substances extracted from this plant species.

Some taxonomists were inspired by this traditional knowledge to name scientific species. A concrete example is the species whose specific epithet is “officinalis, -e,” since this Latin term is translated as an “official product, which comes from the pharmacy,” referring to medicines sold in pharmacies in the past. Therefore, *Salvia officinalis* L., *Melissa officinalis* L., and *Calendula officinalis* L., among many others, are European medicinal species and have been known as such since the 18th century since they were described by Linneaus, who lived in Europe in that century. Another example from Brazil is the Pantanal Matogrossense plant *Heteropterys aphrodisiaca* O. Mach. (popularly known as dog knot), indicated as an aphrodisiac by African descendants [7]. Both the scientific and the vernacular names of this plant refer to this biological activity. Thus, the specific epithet “aphrodisiaca” is translated as “aphrodisiac,” yet the vernacular name of the species “dog knot” alludes to the canine penis after intercourse since the roots of this plant resemble the shape of a penis under such condition.

Despite this standardization of academic science, human populations tend to classify the world according to their own languages and cultures [8]. According to Rodrigues and Barnes [9, 10], among the Krahô people, there are plants called caprãnkohiréhô; *caprãn* means “turtle,” *kohiré* “vertebra,” and *hô* “leaf/plant.” The pharmacological effect of these plants refers to the “slowness of a turtle.” In fact, these plants are used in the form of cigarettes by these indigenous people in search of anxiolytic effects. The most interesting thing about these plants is the way indigenous people acquire knowledge of their effects. They observed that deer, one of the fastest animals in the Cerrado biome, after consuming this plant exhibits altered behaviour, becoming “slower” than usual. In this sense and because they made this observation, they began to apply this plant in different ways until they reached the cigarette, which has a relaxing and anxiolytic effect.

Furthermore, the plant *Dysphania ambrosioides* (L.) Mosyakin & Clemants (Amaranthaceae) is an example of how the colonization process changed vernacular plant names in Brazil. In Ilhabela, a city on the coast of the state of São Paulo, this plant is known as caanema by fishermen communities, which in the Tupi language, “caa” means leaves and “nema” fetid. This name is an inheritance of the Tupinambá population, which occupied the Brazilian coast in the past. Today, this plant species has other names throughout Brazilian territory; erva-de-Santa-Maria (southeast of the country), mastruz (north), and mastruço (several locations). From this example, it is possible to notice that several plants named in native languages changed with the arrival of Europeans, and many of the names paid homage to saints such as Santa Maria.

New bioactive compounds were searched using approaches such as (i) random collections; (ii) chemotaxonomy; (iii) chemical ecology; (iv) zoopharmacognosy, and last, (v) ethnopharmacology/ethnobotany [11–13]. In addition to these methodologies, we suggest a new approach based on the search for clues and indications about plant uses and properties in vernacular and scientific names. This approach alone and/or combined with the others mentioned above could increase our chances of achieving success in the search for new bioactive molecules. Would this not be a universe of possibilities little explored in addressing traditional and vernacular knowledge? It is important to recall that this knowledge was used to “baptize” many genera and epithets, which, in fact, can give us important pharmacological clues of a given specimen, which up until now may have been poorly explored. In addition, although they are often included in the composition of scientific names, are lexicons not overlooked, and thus poorly understood?

In this way, both the scientific (genus name and the specific adjective/epiphyte) and vernacular names of species can be rich sources of data on their possible biological properties/toxicity, guiding chemical and pharmacological research aimed at the development of new drugs. Based on the reading of the two classical and reference works on Brazilian medicinal plants, this study sought to list the scientific and vernacular names of plants whose lexicons are related to the possible medicinal and/or toxic properties of plants and to compare them with the “ethno” (ethnobotanical and ethnopharmacological) and pharmacological data available in the scientific literature.

## 2. Materials and Methods

### 2.1. Books Consulted and Selection of Species

**Figure 1:**
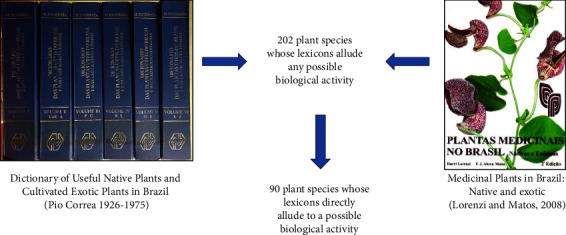
The two literary works from which the 202 plant species were extracted. The 90 plant species whose lexicons directly allude to a possible biological activity are analyzed in the present study.

To carry out this study, two classic and reference works on Brazilian medicinal plants were selected, namely, “Dicionário das plantas úteis do Brasil e das exóticas cultivadas” (Dictionary of Useful Native Plants and Cultivated Exotic Plants in Brazil) by Pio Correa [14] and “*Plantas medicinais no Brasil. Nativas e exóticas*” (Medicinal Plants in Brazil. Native and Exotic) by Lorenzi and Matos [15] (Figure 1). The former consists of six volumes, describes approximately 10,000 plant species, and is considered one of the most important and comprehensive publications on the subject in Brazil; the latter, although more modest in relation to the number of species, identifying approximately 2,800 vernacular names, was included in this study due to its recognized importance [16].

When consulting these books, based on fluctuating reading, that is, based on generic capture of information [17], we searched for native and exotic plants whose scientific and/or vernacular names refer to any biological activity (medicinal and/or toxic). After selecting plant species, new bibliographic surveys were conducted on a scientific basis, and the following data are organized in Table 1: taxonomic family; currently accepted scientific name; scientific name as found in the literature; vernacular name if found (original in Portuguese and translated into English); possible biological properties according to the meaning of the epithets and/or vernacular names of the plant; literature related to ethnobotany/ethnopharmacology (here called “ethno”); and pharmacology. Table 1 presents the data for each plant species that refers to the following three dimensions: possible medicinal and/or toxic properties according to their lexicons, “ethno,” and pharmacological data from the scientific literature.

The scientific names of the species and their families were confirmed using the World Flora Online database (https://www.worldfloraonline.org/). Both the scientific name found in the literature and the currently accepted scientific name according to this database were included. Subsequently, the geographical origin was verified through Flora do Brasil 2020 (https://floradobrasil.jbrj.gov.br/reflora/listaBrasil/PrincipalUC/PrincipalUC.do#CondicaoTaxonCP); native species are marked with the notation (♦) in Table 1.

### 2.2. Bibliographic Survey and Correlations

Correlations between the meanings of the species' epithets and their possible biological properties were made from their translation from Latin by consulting online Latin dictionaries (https://www.dicio.com.br/vermiculares/, https://www.infopedia.pt/dicionarios/termos-medicos/CASTRENS), Vocabulário Latim-Português-Lingua Latina [171], and other bibliographies such as Brown [172], Gledhill [6], and Stearn [173]. Therefore, for example, in the case of *Allamanda cathartica* L., the specific epithet *cathartica* was used as a reference for the correlation mentioned, as its Latin translation is “cathartic,” that is, laxative activity. The correlation of vernacular names with possible biological activities was determined by the actual meaning of the word in Portuguese. Therefore, for example, the poisonous herb plant already contains the term “poisonous.” In Table 1, the terms (specific epithet and vernacular name) that were used in this translation are presented in bold text to facilitate visualization.

**Figure 2:**
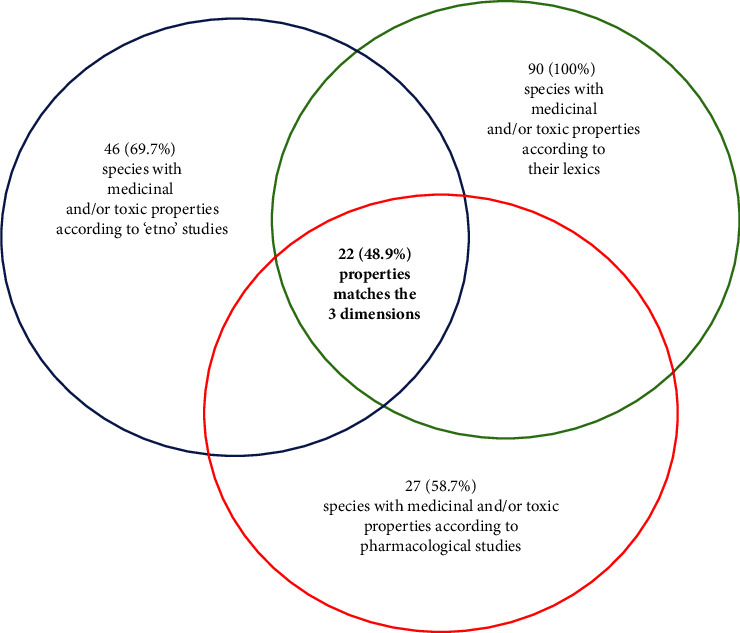
Number and percentage of species that had equivalence in the three dimensions analyzed (lexicon, “ethno,” and pharmacology).

To compare the aforementioned correlations with the “ethno” and pharmacological data present in the scientific literature, bibliographic surveys of various databases (Scopus, PubMed, Scielo, Google Scholar, Science Direct, and others) were conducted in December 2022. In the pharmacological survey, searches involving the scientific names of the plants and terms such as “medicinal,” “toxic,” “toxicity,” and “treatment” were used. The terms “ethnobotany,” “ethnopharmacology,” “traditional knowledge,” and “vernacular name” were used in the ethnobotanical/ethnopharmacological survey, and the search results were limited to English, Spanish, and Portuguese. The “ethno” and/or pharmacological data that coincide with the possible biological properties, lexicon of the plants, are highlighted with asterisks (^*∗*^) in Table 1.  Figure 2 shows these data schematically.

### 2.3. Categorization and Comparison of the Data

**Figure 3:**
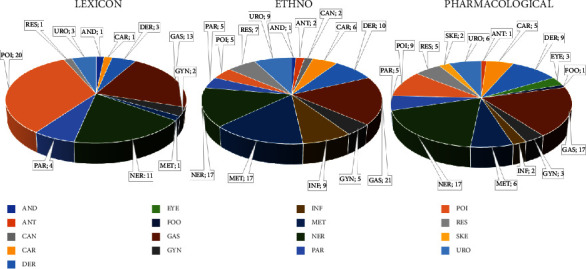
The 17 categories of bioprospecting classification^*∗*^ considering each species in their three dimensions: possible biological property (according to the lexicon of the plant), ethnobotanical/ethnopharmacological, and pharmacological data. ^*∗*^Bioprospecting classification: andrology (AND), antidote (ANT), cancer (CAN), cardiovascular diseases (CAR), dermatologic disorders (DER), ophthalmic problems (EYE), food (FOO), gastrointestinal problems (GAS), gynecology (GYN), infections (INF), metabolic syndromes (MET), nervous system (NER), parasites (PAR), poisons (POI), respiratory complaints (RES), skeletomuscular system (SKE), and urology (URO).

The categorization of the data on the possible biological properties, according to the scientific/vernacular names of plants (lexicons), the “ethno” data, and the pharmacological data on large therapeutic groups in biomedicine was based on the categorization called bioprospection that was proposed by Staub et al. [174]. Accepted by the World Health Organization, this classification proposes 17 broad categories of appropriate use for the discovery of new drugs: antidote (ANT), andrology (AND), cancer (CAN), cardiovascular diseases (CAR), dermatologic disorders (DER), ophthalmic problems (EYE), food (FOO), gastrointestinal problems (GAS), gynecology (GYN), infections (INF), metabolic syndromes (MET), nervous system (NER), parasites (PAR), poisons (POI), respiratory complaints (RES), skeletomuscular system (SKE), and urology (URO). Finally, the categories identified for these three dimensions (lexicons, “ethno,” and pharmacological data) are compared in Figure 3.

## 3. Results and Discussion

Based on the bibliographic survey conducted herein, 202 plant species were found to exhibit some pharmacological properties considering the inherent characteristics of their binomial identification (Figure 1). One hundred and twelve (112) of them, although denoted some medicinal use, was not explicit on which category of bioprospection they could be related. Therefore, for example, *Melissa officinalis* L., whose specific epithet “officinalis” means “medicinal,” could not be categorized in any of the bioprospection classifications. For this reason, these species are not presented in Table 1 but will be addressed later in this article.

On the other hand, the other 90 species had scientific/vernacular names (lexicons) highly related to direct biological or pharmacological activity. They are presented in Table 1 and belong to 47 families; Compositae and Fabaceae (5 species each) being the predominant families, followed by Bignoniaceae, Euphorbiaceae, and Rutaceae (4 species each).

Furthermore, it was observed that of 90 species, 56 (62.2%) are native to Brazilian territory and are identified by the sign “♦” in Table 1. Of the 90 species, 73 lexicons indicate some type of medicinal/toxic property according to their genus/epithet, while 19 lexicons make this allusion according to their vernacular names. These names are shown in bold in Table 1. The epithets *vermicularis*, *anthelminticum*, *antilethargica*, *toxicaria*, *febrifuga*, *cathartica*, *diuretica*, *parasiticus*, *antihysterica*, and *antisyphilitica*, among others were found in the current study (Table 1). In a study conducted by Hecklau et al. [5], the authors observed that most of the specific epithets used in the names of angiosperms in central French Guiana resemble some biological activity; they are *officinalis*, *ophthalmia*, *pectoralis*, and *toxifera*, among others.

Table 1 shows that 66 of the 90 species (73.3%) presented “ethno” data from the scientific literature, while 46 species, or 51.1%, presented pharmacological data. Of the 66 species with “ethno” data, 46 species (69.7%) showed equivalence with the possible medicinal and/or toxic properties of plants according to the plant lexicons (Figure 2), furthermore, of the 46 selected only 27 species (58.7%) exhibited the equivalence between pharmacological data with their lexicons. However, of the 90 species, only 45 had scientific studies in both the “ethno” and pharmacological dimensions, and 22 of them (48.9%), almost half, had equivalence in the three dimensions (lexicon, “ethno,” and pharmacology) (Figure 2). Species with such equivalences are highlighted with asterisks (^*∗*^) in Table 1. Thus, for example, *Eugenia dysenterica* DC. (Myrtaceae) was classified as having possible gastrointestinal biological properties. Data from the “ethno” literature indicate diarrhoea and for pharmacology, therapeutic benefits in recovery from chronic constipation, and irritable bowel syndrome [125, 175]; because all these sources refer to the category of gastrointestinal problems category (GAS) and/or have similarities in the symptoms and disease, these three dimensions are highlighted with asterisks in Table 1.

Table 1 shows and Figure 3 compares the distribution of the 17 categories, bioprospecting classification [174], considering each species in their three dimensions, i.e., lexicons, “ethno,” and pharmacological data; in some cases, the same species was classified in more than one category. For possible biological properties, according to the lexicons of plants, 11 categories were observed; the most frequent being poisonous (20 species), gastrointestinal problems (13), and nervous system (11). The “ethno” data were organized into 14 categories, the most prevalent being gastrointestinal problems (21), nervous system (17), metabolic syndromes (17), and dermatological problems (10). Similarly, pharmacological data were distributed into 15 categories: nervous system (17), gastrointestinal problems (17 species), and dermatologic and poisonous problems (9 species each).

By comparing the three dimensions of the data (Figure 3), the lexicon differs from the “ethno” and pharmacological data, while the last two show greater similarity considering the number of categories and species per category of bioprospecting. This may be related to the fact that many “ethno” and pharmacological studies have been conducted with these species, providing more scientific data compared to the lexicon, which generally has only one possible data. Furthermore, the gastrointestinal and nervous system categories are among the most recurrent in the 3 dimensions. The parasites category was consistent across the 3 dimensions. The category of poisons was the most frequent in the lexical dimension, but these species presented other data from “ethno” and pharmacology and, therefore, in these last two dimensions, this category was diluted among others.

The Compositae family includes plants of different sizes and cosmopolitan distribution and is also one of the largest families of angiosperms, with approximately 1600 genera [124]. Many species have been commercialized for medicinal, ornamental. and culinary purposes, in addition to species known to be invasive in our country [176]. In Table 1, although *Achillea ptarmica* L. has no “ethno” and/or pharmacological studies in the scientific literature, its genus *Achillea* refers to its use by Achilles “to staunch wounds,” and for this reason, it has been categorized as possibly having properties to treat dermatologic disorders (DER). Furthermore, its epithet *ptarmica*, which can be translated as “causing sneezes,” was classified as a plant with biological properties related to respiratory complaints. Other species belonging to the *Achillea* genus, but not surveyed in the present study, such as *A*. *millefolium* L., show agreement between the three dimensions analyzed here. Therefore, *Achillea millefolium* L., popularly known as atroveran and novalgine, has “ethno” records on its use in wounds, as an antipyretic and analgesic [177, 178], while its pharmacological activity includes anti-inflammatory, antipyretic, and analgesic effects [179]. In addition, these vernacular names are the same as two synthetic drugs, Novalgine® (antipyretic and analgesic) and Atroveran® (antispasmodic, used for cramps and pain during menstruation), since according to traditional knowledge, the effect of this species resembles them. As can be seen in Table 1, *Tanacetum parthenium* (L.) Sch. Bip. (Compositae), previously named *Parthenium matricaria* Gueldenst., was classified as gynecology (GYN) since its epithet *matricaria* means *“Mothercare”* (former medicinal use in the treatment of uterine infections) and *parthenium* mentions labor. According to Pareek et al. [180], it has been used by traditional communities in the treatment of infertility and problems with menstruation or during childbirth, while pharmacological investigations showed its effects in the treatment of smooth muscle spasms and as a uterine stimulant. Thus, this species shows an agreement between the three dimensions analyzed here. *Solidago chilensis* Meyen, previously named *Solidago vulneraria* Mart. ex Baker (Compositae), has the ephitet *vulneraria* meaning wound healing property. “Ethno” studies recorded its anti-inflammatory property [78], while pharmacological studies describe its use as an antiulcerogenic [70]. Finally, Table 1 shows that the species *Xanthium catharticum* Kunth (Compositae) alludes to cathartic activity; however, no studies were found in the “ethno” and pharmacological literature to investigate this possible use. However, the species *Allamanda cathartica* Schrad. (Apocynaceae), whose possible property is cathartic according to the lexicon, may be purgative, according to pharmacological studies [71], and has been recorded as a cathartic plant during an ethnobotanical survey [56].

As one of the largest families of angiosperms, Euphorbiaceae currently includes approximately 8,000 species distributed in 317 genera. Found all over the world, these species stand out economically in food and medicine, according to popular knowledge [50]. In Table 1 the species *Cnidoscolus urens* (L.) was categorized as poisons (POI) since both its epithet *urens* and its vernacular name *queimadeira*, resembles the effects “acrid, stinging, burning, to burn.” García et al. [181] confirmed the poisonous activity from a pharmacological study. Table 1 also shows species belonging to the genera Euphorbia and Jatropha, which have many other examples from the literature, such as *Euphorbia tirucalli* L., also known as *Devil's Finger*, which produces a toxic and caustic latex that can cause allergic reactions [87] and *Jatropha curcas* L. which has seeds rich in toxic oil [182]. *Jatropha cathartica* Terán & Berland. is another example; its seeds can also cause nausea, vomiting, diarrhoea, and suppression of intestinal functioning, and a single seed can cause severe poisoning [183]. In Table 1, the species *Croton antisyphiliticus* Mart. has been categorized as infections (INF) since its epithet resembles the antisyphilitic activity. In fact, ethnobotanical studies show similar data, such as syphilis, to treat genital infections and venereal cancers [89, 90, 184].

The species *Leonurus cardiaca* L. (Lamiaceae) shows the relationship between the three dimensions analyzed here. Its epithet *cardiaca* brings the idea of “heart conditions.” In fact, ethnobotanical and pharmacological studies showed symptoms and effects correlated with the heart [91, 103]; in this sense, the species has been categorized as cardiovascular diseases (CAR). The vernacular name *batata-aipo-de-purga* (*Operculina macrocarpa* (L.) Urb.-Convolvulaceae), where *purga* brings the idea of purgative, has been studied by ethnobotanical and pharmacological fields and both confirm its purgative activity [82, 102], and the species has been classified as for gastrointestinal problems (GAS). As can be seen in Table 1, the species *Strychnos nux-vomica* L. (Loganiaceae) has both medicinal and toxic possibilities since *Strychnos* means poisonous from solanaceous plants; while *nux-vomica* means an emetic nut. We know that the difference between medicine and poison is subtle and that many properties that are apparently harmful can be used therapeutically, as indicated, for example, by the epithets *nux vomica*, whose emetic property has been recorded in ethnobotanical studies [81], and the toxic property in pharmacological surveys [108]. In the literature, a clear example of this is *Physostigma venenosum* Balf., which, although it has the specific epithet *venenosum*, has already been used as a medicine (physostigmine) in the treatment of Alzheimer's disease [109].

In Table 1, we can observe other plants that have “ethno” studies equivalent to their lexicons and could be studied pharmacologically as follows: *Uvaria febrifuga* Humb. & Bonpl. ex DC. (Annonaceae) as febrifuge; *Urtica urens* L. (Urticaceae), which causes phytodermatoses; *Richardsonia emetica* Mart. (Rubiaceae) as emetic; *Polygonum antihaemorrhoidale* Mart. (Polygonaceae) as an antihemorrhoid; and *Turnera aphrodisiaca* Ward (Passifloraceae) as an aphrodisiac, among others. Other plants have “ethno” studies equivalent to their lexicons but do not have pharmacological data; *Ilex diuretica* Mart. ex Reissek (Aquifoliaceae) as a diuretic; *Luffa purgans* (Mart.) Mart. (Cucurbitaceae) as a purgative; *Aristolochia theriaca* Mart. ex Duch (Aristolochiaceae), with antiophidic action; *Andira vermifuga* Benth. (Fabaceae) as a vermifuge; and *Lobaria pulmonaria* (L.) Hoffm. (Lobariaceae) for pulmonary disorders, among others. In the same table, we observe some other species that have no studies in the scientific literature (“ethno”/pharmacology), only the lexical indication, and therefore, they and their respective potentialities should be the target of future studies as follows: *Pothos parasiticus* Vell. (Araceae), *Rhipsalis parasitica* (Lam.) Haw. (Cactaceae), *Anthurium parasiticum* (Vell.) Stellfeld (Araceae), *Cactus parasiticus* L. (Cactaceae), and *Momordica anthelmintica* Schumach. & Thonn. (Cucurbitaceae) as possible antiparasitics; *Xanthium catharticum* Kunth (Compositae) and *Trimezia cathartica* (Klatt) Niederl. (Iridaceae) as cathartics; *Ecballium purgans* Schrad. (Cucurbitaceae) as purgative; and *Nesaea syphilitica* (DC.) Steud (Lythraceae) as an anti-infectious agent, among others.

In addition to the 90 species listed in Table 1, another 112 were found in the works analyzed here. Although they do not show a direct correlation between their names (genus, epithet, and vernacular name) and a possible biological activity, they present exciting names from the medicinal and/or toxic point of view. In this sense, these 112 species will be presented and discussed in the following.

### 3.1. Correlation of Vernacular Names and Biological Properties

In the present work, nine vernacular names that indirectly refer to a possible biological activity were found in this search. Plants whose vernacular names in Portuguese include the word “*diabo*” (devil), such as *jarro-do-diabo* (devil's pitcher), whose scientific name is *Aristolochia cymbifera* Mart. & Zucc. (Aristolochiaceae), indicating a negative property. This vernacular name suggests “*a pitcher belonging to the devil*,” most likely due to its toxicity. This plant, in particular, is known for its carcinogenic and nephrotoxic properties as a member of the Aristolochiaceae family [185]. Other species in this category include *herva-do-diabo* (devil's herb), *Elephantopus mollis* Kunth. (Compositae), and *café-do-diabo* (devil's coffee), *Casearia guianensis* (Aubl.) Johnson (Salicaceae).

Other vernacular names stand out, such as *sanguinaria* (sanguinary), *Persicaria acuminata* (Kunth) M. Gómez (Polygonaceae), which may refer to an antihaemorrhagic or, conversely, haemorrhagic action. Finally, herva-almiscar-dos-feridos (musk herb of the wounded), *Canna glauca* L. (Cannaceae), refers to positive benefits for injured people, without further details.

All of the abovementioned names are vague, precluding a correlation between them and possible biological activity despite providing some type of information on medicinal properties and/or toxicity.

### 3.2. Correlation of Genera and Biological Properties

In this work, 51 species whose genera refer to medicinal uses and/or toxicity were recorded. Translations of these terms for possible medicinal and/or toxic potentials were based on readings of Latin dictionaries, as described in the Methods section. Many of them refer to historical facts in medicine, including the genus *Herniaria* of *H. paico* Molina (Amaranthaceae), which means “hernia rupture,” a term previously used in medicine, as well as *Angelica archangelica* L. (Apiaceae). In fact, a review shows us dozens of biological activities attributed to this plant from contemporary studies [186], whose genus means “healing power.” *Consolida major* Garsault (Boraginaceae) is an old Latin name used for “curative drugs.” *Matricaria proealta* (*Matricaria praealta* Poir.) (Compositae) refers to “maternal care” (previously used in medicine to treat uterine infections). Furthermore, various genera refer to toxic properties, such as *Cerbera triphylla* Rudge (Apocynaceae), a genus referring to the term Cerberus, which means poisonous, and *Toxicodendron divaricatum* Greene, where Toxicodendron means poisonous tree. The term *Aethusa* of the species *A. cynapium* L. means “burning, due to its pungency.”

### 3.3. Correlation of Epithets and Biological Properties

In total, 52 species were found in this search, with the following epithets: *officinalis*, *officinale*, *officinarum*, *medica*, *medicinalis*, *salutaris*, or *ipecacuanha*. All of these terms refer to some medicinal activity, albeit without a specific reference.

The terms *officinale* and *officinarum* can be translated as synonyms of *officinalis* and have the same meaning. Most, or more specifically, 90% of these 52 species have one of these three epithets. In fact, some of these species have already been widely studied and their medicinal activities have been established, for example, *Melissa officinalis* L., which reduces depression, anxiety, and stress and acts on sleep disorders [187]; *Mikania officinalis* Mart. as an anti-inflammatory, analgesic, and antibacterial [188]; and *Calendula officinalis* Hohen. as anti-inflammatory [189].

Three other species have the epithets *medica* or *medicinalis*, namely, *Ilex medica* Reissek (Aquifoliaceae), *Psychotria medica* Müll. Arg. (Rubiaceae), and *Simarouba medicinalis* Endl. (Simaroubaceae), which refers to medicinal use, although without specifying the possible effect. The epithet *salutaris* of the species *Vitis salutaris* (Kunth) Baker (Vitaceae) also refers to its beneficial use. Finally, the ipecacuanha epithet of the *Evea ipecacuanha* (Brot.) species W. Wight (Rubiaceae) means “drug producer” and may be related to some medicinal activity.

### 3.4. More than One Term Indicating Some Biological Activity

Some names are related to biological activity in more than one term (genus/epithet/vernacular name). Therefore, in the case of the species *Avicennia officinalis* L., both terms contain this information and *Avicennia* refers to Avicena (980–1037), an Arabic philosopher and physician, while its epithet *officinalis* has already been discussed in the previous section. *Valeriana officinalis* L., whose genus means “health (valere),” was named in medieval times for its medicinal use. *Artemisia absinthium* L., whose genus means “female pains,” refers to the Goddess Artemisia and its epithet *absinthium* is a Greek term that refers to the aromatic herb used in medicine. *Althaea officinalis* L., whose genus mentions “healer,” was named by Theophrastus.

### 3.5. Limitations of the Present Study

One of the limitations of the present study is the translation of the Latin term “gender” and/or “specific epithet” into a possible biological property. For example, the epithet *lumbricoides* may be associated with the worm format (roundworm) but also with some biological activity related to this worm, such as being worm-like. The same is true for *Ilex fertilis* Reissek and *Avena sterilis* L., epithets related to plant fertility and sterility, respectively. However, these characteristics could be as closely related to the characteristics of the plant as to its use. Thus, “ethno” and pharmacological studies can help clarify the possible properties of plant lexicons.

However, the intended analysis, comparing the possible biological property of the plant from its lexicon with ethno and pharmacological studies, was hampered since few “ethno” and pharmacological studies were located in the scientific literature, 66 and 46 in 90 plants, respectively. The pharmacological studies found in the scientific literature were not based on the possible biological activities investigated here, that is, the fact that the species *Blutaparon vermiculare* (L.) Mears has not been investigated for possible worming activity does not mean that it does not have this activity. Finally, most of the time, the lexicon refers only to a possible biological activity, while many uses can be attributed to the same species, both from an “ethno” and a pharmacological point of view. All these factors limited the analysis of this study, but despite them, a high percentage of coincidence was observed considering the three dimensions for 48.9% of the investigated plants.

### 3.6. Future Perspectives

The data collected in the present study favor new bioprospecting investigations that consider, in addition to the approaches already used (at random; collecting orientated by chemotaxonomy; biorational collecting, guided by chemical ecology; and collecting based on traditional knowledge, known as ethnopharmacology), another approach that basically considers plant lexicons. The sum of these approaches when choosing a future plant species to be tested by pharmacology and phytochemistry can and should increase the chances of achieving success in the search for new bioactive potentials.

## 4. Conclusions

In the present review, two classical Brazilian books on native and exotic plants were consulted and based on both, 10,394 plant species were collected and analyzed by fluctuating reading, allowing to associate scientific and vernacular names with biological activity. Furthermore, it was observed that according to the classic literature of medicinal plants, a high rate of concordance between scientific names and biological activity was observed, which was further validated by elegant ethnopharmacological and pharmacological studies. According to this study, 22 (48.9%) of the 45 plants surveyed showed equivalence in the three dimensions (lexicon, “ethno,” and pharmacology). We, therefore, conclude that plant lexicons may be closely linked to the possible medicinal and/or toxic properties of plants, and thus it is conceivable that plant lexicons may represent one additional approach for the characterization of new drugs. Therefore, in addition to data extracted from ethnopharmacology, random, or chemotaxonomy, lexicons may represent an interesting method to guide medicinal plant research. Therefore, based on a robust number of analyzed species, it was possible to conclude that plant lexicons were correlated with scientific names and, more importantly, with medicinal, pharmacological, or toxic properties. Finally, lexicons may represent an important approach for the search of new drugs, mainly directed to gastrointestinal, nervous, and parasites diseases.

## Figures and Tables

**Table 1:** Ninety species grouped by family, currently accepted name, name found in the literature, vernacular name, possible medicinal and/or toxic properties, ethnobotanical/ethnopharmacological data (“ethno”), and pharmacological data (both obtained from the scientific literature).

Family	Currently accepted name	Species found in the literature	Vernacular name	Possible medicinal and/or toxic properties	“ETHNO” data (ethnobotany/ethnopharmacology)	Pharmacological data
**Achariaceae** (2 species)	Asclepias campestris Vell.	*Asclepias curassavica* L.	**Cega-olhos**	POI (**cega-olhos** = blind eyes)^*∗*^	May cause corneal edema^*∗*^, used for fever, bleeding control, and as a purgative [18–23]	Anti-inflammatory, analgesic, antipyretic, antimicrobial, cytotoxic, and cardiovascular properties [24]
	*Carpotroche brasiliensis* (Raddi) A Gray♦	*Carpotroche brasiliensis* (Raddi) A. Gray	**Fructa-de-arara-de-lepra**	INF (**lepra** = Hansen's disease)^*∗*^	Antileprosy^*∗*^ [25]	Anti-inflammatory and antinociceptive properties [26]
			**Herva-de-piolho**	DER (**herva-de-piolho** = herb of lice)^*∗*^	Treatment of skin conditions^*∗*^ [27]	

**Amaranthaceae** (3 species)	*Blutaparon vermiculare* (L.) Mears♦	*Gomphrena * **vermicularis L**.		PAR (**vermicularis** = worm-like)^*∗*^	Dysentery^*∗*^, diarrhoea^*∗*^, stomach and kidney problems [28]	ND
	*Dysphania ambrosioides* (L.) Mosyakin & Clemants	*Chenopodium * **anthelminticum L**.		PAR (**anthelminticum** = vermifuge, worm expelling)^*∗*^	Antiparasitic ^*∗*^, analgesic, antiasthmatic, digestive, and antihemorrhoidal activities [29–32]	Antiparasitic, trypanocidal, and antileishmanial activities^*∗*^ [33]
	*Gomphrena antilethargica* Silveira	*Gomphrena * **antilethargica** Silveira		NER (**anti-lethargica** = antilethargic)	ND	ND

**Amaryllidaceae** (3 species)	*Boophone disticha* (L.f.) Herb.	*Brunsvigia * **toxicaria** (L.f. ex Aiton) Ker Gawl.		POI (**toxicaria** = toxic, containing a poisonous substance)^*∗*^	hallucinations^*∗*^, stupor and coma^*∗*^, hysteria, headaches, used for boils, burns and abdominal pain [34–37]	Depressive effects on the central nervous system^*∗*^, can cause seizures and difficulty breathing^*∗*^ [38]
	*Crinum asiaticum* L.	*Crinum * **toxicarium** Roxb.		POI (**toxicarium** = toxic, containing a poisonous molecule)	Edema, fever, bruises, fractures, rheumatism, earache, mumps, tonsillitis, gastrointestinal complaints, hernia, to treat vomiting and urinary difficulties [39–41]	Antioxidant, anti-inflammatory, analgesic, cytotoxic, and antimicrobial activities in wound healing [42]
	*Haemanthus toxicarius* L.f. ex Aiton	*Haemanthus * **toxicarius** L.f. ex Aiton		POI (**toxicarius** = toxic, containing a poisonous molecule)^*∗*^	Urinary diseases, skin diseases, swelling, hematoma, respiratory problems, gastrointestinal problems, used as a dart poison^*∗*^ [43, 44]	Antimicrobial, anti-inflammatory, causes hallucinations^*∗*^, treatment for anxiety, stress, and Alzheimer's disease [45]

**Anacardiaceae** (2 species)	*Persea caustica* Spreng.	*Persia * **caustica** = *Persea caustica* Spreng.		POI (**caustica** = with caustic taste, mouthburn)	ND	ND
	*Toxicodendron pubescens* Mill.	*Rhus * **toxicarium** Salisb.		POI (**toxicarium** = toxic, containing a poisonous molecule)^*∗*^	Causes skin irritation^*∗*^, used for swelling, antiseptic activity, used as poison^*∗*^ [46]	Protection against inflammatory lesions, hematologic alterations, and arthritis pain [47]
		*Rhus * **toxicodendron** L.		POI (**toxicodendron** = poison tree)^*∗*^		

**Annonaceae** (1 species)	*Xylopia aromatica* (Lam.) Mart. ♦	*Uvaria * **febrifuga** Humb. & Bonpl. ex DC.		MET (**febrifuga** = fever-dispelling)^*∗*^	malaria^*∗*^ and fever^*∗*^ [48].	*in vitro* activity against *Plasmodium falciparum*^*∗*^ [49]

**Apocynaceae** (3 species)	*Allamanda schottii* Pohl ♦	*Allamanda * **cathartica** Schrad.		GAS (**cathartica** = purgative, purging, cathartic)^*∗*^	Cathartic^*∗*^, emetic, colic ^*∗*^, antifungal and antiviral activities, used to treat fever, diabetes, reduces inflammation, diuretic, and increases blood flow [50–55]	Compounds with analgesic, anti-inflammatory, and antidiabetic activities, cause infertility, purgative^*∗*^ and wound healing [56]
	*Aspidosperma illustre* (Vell.) Kuhlm. & Pirajá♦	*Aspidosperma illustre* (Vell.) Kuhlm. & Pirajá	**Erva-venenosa**	POI (**erva-venenosa** = poison weed)	ND	ND
	*Schubertia multiflora* Mart.	*Schubertia multiflora* Mart.	**Maria-da-costa- peidorreira**	GAS (**peidorreira** = farting)	Abortive and emmenagogue [57]	ND

**Aquifoliaceae** (1 species)	*Ilex diuretica* Mart. ex Reissek♦	*Ilex * **diuretica** Mart. ex Reissek		URO (**diuretica** = diuretic)^*∗*^	diuretic^*∗*^ and used for urinary infections, used for high blood pressure and wound healing [58]	ND

**Araceae** (1 species)	*Anthurium parasiticum* (Vell.) Stellfeld ♦	*Pothos * **parasiticus** Vell.		PAR (**parasiticus** = living at the expense of another, parasitic)	ND	ND

**Aristolochiaceae** (2 species)	*Aristolochia theriaca* Mart. ex Duch. ♦	**Aristolochia theriaca Mart. ex Duch**.		GYN (**Aristolochia** = best-childbirth, abortifacient property)/ANT (**theriaca** = antidote, theriacs are antidotes to poisons and bites of wild beasts)^*∗*^	Antiophidic action^*∗*^ [59]	ND
	*Aristolochia triangularis* Cham. ♦	**Aristolochia antihysterica** Mart. ex Duch.		GYN (**Aristolochia** = abortive)/NER (**antihysterica** = antihysteria)^*∗*^	Hysteria attacks^*∗*^, soothing nerves ^*∗*^, antiseptic use, infections, skin diseases, ulcers, diuretic, edema, malaria, parasites, hypertension, dysentery, diarrhoea, abortive^*∗*^ and antirheumatic [27, 60, 61]	Anti-inflammatory activity [61]

**Bignoniaceae** (4 species)	*Cybistax antisyphilitica* (Mart.) Mart. ♦	*Bignonia * **antisyphilitica** Mart.		INF (**syphilitica** = to treat syphilis)^*∗*^	Antisyphilitic activity, lotions against syphilitic ulcers^*∗*^, decoction and infusion to treat dysuria, swelling, and water retention [62]	Larvicidal activity against *Aedes aegypti* [63]
		*Cybistax * **antisyphilitica** (Mart.) Mart.				
		*Phryganocydia * **antisyphilitica** Mart. ex DC.				
	*Handroanthus barbatus* (E.Mey.) Mattos ♦	*Couralia * **toxophora** (Mart.) Benth. & Hook.f. ex K.Schum.		POI (**toxophora** = toxic, containing a poisonous substance)	Antimalarial activity [64]	ND
	*Handroanthus impetiginosus* (Mart. ex DC.) Mattos ♦	*Tecoma impetiginosa* Mart.	**ipê-contra-sarna**	DER (**sarna** = scabies)^*∗*^	Fever reducer, venereal and rheumatic disorders, for skin disorders (eczema, herpes, and mange) and ulcers^*∗*^ [64]	Anti-inflammatory, antiautoimmune activities ^*∗*^, and anticancer [60, 65, 66]
	*Kordelestris syphilitica* Arruda	*Kordelestris * **syphilitica** Arruda		INF (**antisyphilitica** = anti- syphilis)	Treatment of skin diseases (scarring) ^*∗*^, respiratory and digestive disorders [67]	ND

**Cactaceae** (2 species)	*Rhipsalis baccifera* (J.S.Muell.) Stearn ♦	*Rhipsalis * **parasitica** (Lam.) Haw.		PAR (**parasitica** = living at the expense of another, parasitic)	ND	ND
	*Rhipsalis undulata* Pfeiff.	*Cactus * **parasiticus** L.		PAR (**parasiticus** = living at the expense of another, parasitic)	ND	ND

**Caryocaraceae** (1 species)	*Caryocar glabrum* (Aubl.) Pers.	*Caryocar * **toxiferum** Barb.Rodr.		POI (**toxiferum** = toxic, containing a poisonous substance)	ND	ND

**Celastraceae** (1 species)	*Monteverdia truncata* (Nees) Biral	*Monteverdia truncata* (Nees) Biral	**Erva-botão-cancerosa/erva-botão-cancrosa**	CAN (**cancerosa**/**cancrosa** = cancerous)	Stomach problems [68]	ND

**Commelinaceae** (1 species)	*Tripogandra diuretica* (Mart.) Handlos ♦	*Tradescantia * **diuretica** Mart.		URO (**diuretica** = diuretic)^*∗*^	Diuretic, urinary^*∗*^, liver, disorders [69].	ND

**Compositae** (5 species)	*Achillea ptarmica* L.	**Achillea ptarmica** L.		DER (**Achillea** = used by Achilles to staunch wounds)/RES (**ptarmica** = causing sneezes)	ND	ND
	*Mikania cynanchifolia* Hook. & Arn. ex B.L.Rob. ♦	*Mikania * **cynanchifolia** Hook. & Arn. ex Baker		POI (**cynanchifolia**- *cynanchicus* = of quinsy, literally dog throttling, from its former medicinal use)	ND	ND
	*Solidago chilensis* Meyen♦	*Solidago * **vulneraria** Mart. ex Baker		DER (**vulneraria** = wounds, healing wounds)^*∗*^	Anti-inflammatory^*∗*^, diuretic, and gastrointestinal disorders [70]	Decreased gastric lesions^*∗*^ [71]
	*Tanacetum parthenium* (L.) Sch.Bip.	**Parthenium matricaria** Gueldenst.		GYN (**matricaria** = maternal care, former medicinal use in treatment of uterine infections; **parthenium** = for labor)^*∗*^	Fevers, rheumatoid arthritis, migraines, toothache, stomach pain, insect bites, infertility, and problems with menstruation or during childbirth^*∗*^ [72–77]	Treatment for smooth muscle spasms, prophylactic treatment for migraine, inhibition of histamine release (inflammatory processes)^*∗*^, uterine-stimulant activities^*∗*^ [78, 79]
	*Xanthium catharticum* Kunth	*Xanthium * **catharticum** Kunth		GAS (**catharticum** = purgative, purging, cathartic)	ND	ND

**Convolvulaceae** (2 species)	*Ipomoea dumosa* (Benth.) L.O. Williams ♦	*Exogonium * **purga** (Wender.) Benth.		GAS (**purga** = purgative)^*∗*^	purgative^*∗*^ [80]	ND
	*Operculina macrocarpa* (L.) Urb.♦	*Ipomoea operculata* Mart. & Spix	**Batata-aipo-de-purga**	GAS (**purga** = purgative)^*∗*^	Purgative^*∗*^, anthelmintic, blood purifier and treatment for uterine infection [81]	It has resin glycosides known as purgative ingredients^*∗*^ [82].

**Cucurbitaceae** (3 species)	*Ecballium elaterium* (L.) A.Rich.	*Ecballium * **purgans** Schrad.		GAS (**purga** = purgative)	ND	ND
	*Luffa sepium* (G.Mey.) C.Jeffrey	*Luffa * **purgans** (Mart.) Mart.		GAS (**purga** = purgative)^*∗*^	Swelling, chronic ophthalmia (eye diseases), and emetic properties [62]	ND
	*Momordica anthelmintica* Schumach. & Thonn.	*Momordica * **anthelmintica** Schumach. & Thonn.		PAR (**anthelmintica** = vermifuge, worm expelling)	ND	ND

**Erythroxylaceae** (1 species)	*Erythroxylum cataractarum* Spruce ex Peyr.♦	*Erythroxylum * **cataractarum** Spruce ex Peyr.		EYE (**cataractarum** = cataract)	The snuff protects people from bad weather, such as cold and rain, and from long, sleep-disturbing activities [83–85]	ND

**Euphorbiaceae** (4 species)	*Cnidoscolus urens* (L.) Arthur♦	*Cnidoscolus * **urens** (L.) Arthur	**Queimadeira**	POI (**urens** = **queimadeira** = acrid, stinging, burning, to burn)^*∗*^	Antiparasitic, to treat boils and “ringworm” (superficial skin mycoses), itching, injuries, skin infections, skin wounds, stomach pain, uterine infections, and blood purification [86]	Contains several toxins, such as curcin (toxalbumin phytotoxin), hydrocyanic acid, atropine, tetramethylpyrazine, glycoside, and curcanoleic acid^*∗*^ [87, 88]
		*Jatropha * **urens** L.				
	*Croton antisyphiliticus* Mart. ♦	*Croton * **antysiphiliticus** Mart.		INF (**antisyphilitica** = anti-syphilis)^*∗*^	Syphilis^*∗*^, rheumatisms, ulcerative lesions, inflammatory diseases, genital infections, and venereal cancers^*∗*^ and to treat infections of the urogenital tract [89–91]	Nematocidal activity *in vitro*^*∗*^ [92]
	*Euphorbia ophthalmica* Pers. ♦	*Euphorbia * **ophthalmica** Pers.		EYE (**ophthalmica** = ophtalmic)	ND	ND
	*Jatropha podagrica* Hook.	*Jatropha * **podagrica** Hook.		MET (**podagrica** = snare, of gout, used to treat gout)^*∗*^	Antipyretic, diuretic, choleretic and purgative, to cure oral candidiasis in infants, to treat worms, skin diseases, and as a wound dressing [93, 94]	Antitumor, antimicrobial, molluscicidal, and insecticidal activities [95]

**Fabaceae** (5 species)	*Andira fraxinifolia* Benth. ♦	*Andira fraxinifolia* Benth.	**Mata-baratas**	POI (**mata-baratas** = insecticide)^*∗*^	Antihelminthic activity ^*∗*^ (the genus *Andira* is still used despite its toxic effects)^*∗*^ [96]	ND
		*Andira * **anthelmintica** Benth.		PAR (**anthelmintica** = vermifuge, worm expelling)^*∗*^		
	*Andira vermifuga* Benth. ♦	*Geoffroea vermifuga* Mart.		PAR (**vermifuga** = vermifuge, worm expelling)^*∗*^	*Andira* species are commonly used for their antihelminthic properties^*∗*^ [97]	ND
		*Andira * **vermifuga** Benth.				
	*Chamaecrista cathartica* (Mart.) H.S.Irwin & Barneby ♦	*Cassia * **cathartica** Mart.		GAS (**cathartica** = purgative, purging, cathartic)^*∗*^	ND	Purgative properties^*∗*^ [98]
	*Chamaecrista nictitans* var. *jaliscensis* (Greenm.) H.S. Irwin & Barneby ♦	*Cassia * **hypnotica** Vell.		NER (**hypnotica** = hypnotic)	ND	ND
	*Vouacapoua vermifuga* (Mart. ex Benth.) Kuntze	*Vouacapoua * **vermifuga** (Mart. ex Benth.) Kuntze		PAR (**vermifuga** = vermifuge, worm expelling)	ND	ND

**Hypericaceae** (1 species)	*Vismia latifolia* (Aubl.) Choisy ♦	*Vismia latifolia* (Aubl.) Choisy	**Pau-alazão-de-febre**	MET (**febre** = antipyretic)^*∗*^	Tonic and febrifugal agent^*∗*^ [99]	Vasodilator effect [100]

**Iridaceae** (2 species)	*Trimezia cathartica* (Klatt) Niederl.	*Cypella * **cathartica** (Klatt) Mart. ex Klatt		GAS (**cathartica** = purgative, purging, cathartic)	ND	ND
		*Lansbergia * **catartica** Klatt				
	*Trimezia juncifolia* (Klatt) Benth. & Hook.f. ♦	*Cypella * **purgans** Mart. ex Klatt		GAS (**purga** = purgative)	For blood clearance, for intermittent wounds and as an anti-inflammatory [101]	ND
		*Ferraria * **purgans** Mart.				
		*Lansbergia * **purgans** Klatt				

**Lamiaceae** (1 species)	*Leonurus cardiaca* L.	*Leonorus * **cardiaca** L.		CAR (**cardiaca** = heart conditions)^*∗*^	Cardiac^*∗*^, nervous system problems, digestive disorders, bronchial asthma, climacteric symptoms, amenorrhoea, skin wounds, and inflammation [102]	Antibacterial, antioxidant, anti-inflammatory, as well as effects on the heart and circulatory system^*∗*^, hypotensive effect^*∗*^, sedative and analgesic activities [103]

**Lobariaceae** (1 species)	*Lobaria pulmonaria* (L.) Hoffm.	*Sticta * **pulmonaria** (L.) Biroli		RES (**pulmonaria** = lungwort, the signature of the spotted leaves as indicative of efficacy in the treatment of respiratory disorders)^*∗*^	Used in pulmonary disorders^*∗*^ [104]	ND

**Loganiaceae** (3 species)	*Spigelia anthelmia* L. ♦	*Spigelia anthelmia* L.	**Lombrigueira/erva-das-lombrigas**	PAR (**lombrigueira; erva- das-lombrigas** = roundword weed)^*∗*^	Anthelmintic^*∗*^ and fish poison [105, 106]	Neuralgic and heart diseases [107]
	*Strychnos nux-vomica* L.	**Strychnos nux-vomica** L.		POI (**Strychnos** = poisonous, solanaceous plants)^*∗*^/GAS (**nux-vomica** = emetic nut)	Eye infection, emetic^*∗*^ and snake bites [108]	Nervous system stimulant, analgesic action, aid in alcohol dependence, anti-inflammatory action, affects the immune system, antitumor activity, snake antivenom, effects on pathogenic microorganisms, toxic^*∗*^ [47, 131]
	*Strychnos pseudoquina* A. St.-Hil. ♦	*Geniostoma * **febrifugum** Spreng.		MET (**febrifugum** = antipyretic)^*∗*^	Abortive [9]	Mutagenic activity (leaves), antimalarial^*∗*^ [9, 111]

**Lythraceae** (2 species)	*Heimia salicifolia* (Kunth) Link ♦	*Ginoria * **syphilitica** Moc. & Sessé ex DC.		INF (**syphilitica** = to treat syphilis)^*∗*^	Antimicrobial activity [112]	Antihypertensive, vasorelaxant effect [113]
	*Nesaea syphilitica* (DC.) Steu	*Heimia * **syphilitica** DC.		INF (**syphilitica** = to treat syphilis)	ND	ND
		*Nesaea * **syphilitica** (DC.) Steud.				

**Malvaceae** (1 species)	*Pavonia sidifolia* Kunth ♦	*Pavonia * **diuretica** A.St.-Hil.		URO (**diuretica** = diuretic)^*∗*^	Emollient, diuretic, and dysuria^*∗*^ [114]	ND

**Melastomataceae** (1 species)	*Miconia tomentosa* (Rich.) D. Don ex DC. ♦	*Micania tomentosa* (Benth.) Fritsch	**Oiti-cagão**	GAS (**cagão** = laxative)	Genital steam baths [115]	ND

**Meliaceae** (1 species)	*Guarea guidonia* (L.) Sleumer ♦	*Guarea * **purgans** A.Juss.		GAS (**purga** = purgative)	Malaria, flu and fever [116]	Wound-healing properties [117]

**Moraceae** (3 species)	*Antiaris toxicaria* Lesch.	**Antiaris toxicaria** Lesch.		POI (**Antiaris** = against association). The Javan upas tree, **Antiaris toxicaria**, reputedly causes the death of anyone who sleeps under it^*∗*^	Antioxidant, anti-inflammatory, febrifuge, antifungal, antibacterial, and for the treatment of dysentery [118]	Anticonvulsant activity, toxic^*∗*^ [119]
	*Ficus adhatodifolia* Schott ♦	Ficus **anthelminthica** Mart.		PAR (**anthelmintica** = vermifuge, worm expelling)^*∗*^	Vermifuge^*∗*^ [120, 121]	Antioxidant and mutagenic activity [122]
		*Ficus * **vermifuga** (Miq.) Miq.				
		*Pharmacosycea * **anthelmintica** (rich. ex DC.) Miq.				
		*Pharmacosycea * **vermifuga** Miq.				
	*Ficus gomelleira* Kunth & C.D. Bouché ♦	*Ficus gomelleira* Kunth & C.D. Bouché	**Gameleira-de-purga**	GAS (**purga** = purgative)^*∗*^	vermifuge and purgative^*∗*^ [114]	Modulator of bacterial activity [123]

**Myrtaceae** (3 species)	*Eugenia dysenterica* DC. ♦	*Eugenia * **dysenterica** DC.		GAS (**dysenterica** = disinteric)^*∗*^	Hookworm (anemia), high blood pressure, inflammation, vaginal discharge, diarrhoea^*∗*^, depurative, diabetes, malaria, and hepatic disease [86]	Antifungal activity, molluscicidal activity, therapeutic benefits in recovery from chronic constipation and irritable bowel syndrome ^*∗*^, anticancer effects; hepatoprotection; antidiabetes; antimicrobials; and cardioprotective [124, 125]
		*Stenocalyx * **dysentericus** (DC.) O.Berg				
	*Eugenia myrcianthes* Nied. ♦	*Campomanesia * **cagaiteira** Kiaersk.		GAS (**cagaiteira** = cathartic)	ND	ND
	*Myrtus dysenterica* Mart. ♦	*Myrtus * **dysenterica** Mart.		GAS (**dysenterica** = disinteric)^*∗*^	Diabetes, malaria, jaundice, and hepatic disease [124]	Anticancer effects, hepatoprotection, antidiabetes, antimicrobials, and cardioprotective [124]

**Passifloraceae** (2 species)	*Passiflora coccinea* Aubl. ♦	*Passiflora * **toxicaria** Barb. Rodr.		POI (**toxicaria** = toxic, containing a poisonous substance)	Psychoactive, hallucinogenic, stimulating, anxiolytic, dysmenorrhea of the genitourinary system, kidney stones, kidney disorders, and fever [126]	ND
	*Turnera diffusa* Willd. ex Schult. ♦	*Turnera * **aphrodisiaca** Ward		NER (**afrodisíaca** = aphrodisiac)^*∗*^	Aphrodisiac, stimulant, nervous tonic^*∗*^, laxative, also acts in kidney, menstrual, and pregnancy disorders [127, 128]	Hypoglycemic activity, aphrodisiac activity^*∗*^ [129]

**Phyllanthaceae** (1 species)	*Phyllanthus urinaria* L. ♦	*Phyllanthus * **urinaria** L.		URO (**urinaria** = diuretic)^*∗*^	Diabetes^*∗*^, malaria, jaundice, and liver disease [130–132]	Anticancer, hepatoprotective, antidiabetic ^*∗*^, antimicrobial, and cardioprotective effects [133]

**Phytolaccaceae** (1 species)	*Microtea debilis* Sw. ♦	*Microtea debilis* Swartz	**Herva-mijona**	URO (**mijona** = diuretic)^*∗*^	proteinuria^*∗*^ [134]	proteinuria^*∗*^ [135]

**Poaceae** (1 species)	*Digitaria setigera* Roth	*Panicum * **pruriens** Trin.		POI (**pruriens** = irritant, stinging, itch-causing)	ND	ND

**Polygonaceae** (1 species)	*Polygonum antihaemorrhoidale* Mart.	*Polygonum * **antihaemorrhoidale** Mart.		GAS (**antihemorrhoidale** = antihemorrhoidal)^*∗*^	Depurative, hemorrhoids, and diarrhoea^*∗*^ [86]	Hemorrhoids^*∗*^ and vermicide [136]

**Ranunculaceae** (2 species)	*Handroanthus barbatus* (E.Mey.) Mattos	*Tecoma * **toxophora** Mart.		POI (**toxophora** = toxic, containing a poisonous substance).	Antimalarial [137]	Cytotoxic activity [138]
	*Helleborus foetidus* L.	**Helleborus ** *foetidus* L.		POI (**Helleborus** = poison-food)	ND	ND

**Resedaceae** (1 species)	*Reseda odorata* L.	**Reseda ** *odorata* L.		DER (**Reseda** = the name, *resedo*, in Pliny refers to its use in treating bruises)^*∗*^	Stomach cramps [139]	anti-inflammatory^*∗*^ and diuretic activities [140, 141]

**Rhamnaceae** (1 species)	*Discaria americana* Gillies & Hook. ♦	*Discaria * **febrifuga** Mart.		MET (**febrifuga** = antipyretic)^*∗*^	Tonic and febrifuge^*∗*^ [142]	Antimicrobial activity [143]

**Rubiaceae** (3 species)	*Palicourea rigida* Kunth ♦	*Palicourea * **diuretica** Mart.		URO (**diuretica** = diuretic)^*∗*^	Kidney disease, inflammation, and urinary tract infections ^*∗*^ and female genital tract, hepatitis, and malaria [144–146]	Anti-inflammatory, antioxidant, antibacterial, and antifungal activities^*∗*^ [147]
	*Richardia brasiliensis* Gomes ♦	*Richardsonia * **emetica** Mart.		GAS (**emetica** = emetic)^*∗*^	Expectorant, diaphoretic, vermifuge, emetic^*∗*^, and to treat hemorrhoids [148].	Emetic^*∗*^, treat hemorrhoids, antidiabetic, vermifuge, eczema treatment, burns, bronchitis, flu, and avian malaria [149, 150].
	*Ronabea emetica* (L.f.) A.Rich. ♦	*Cephaelis * **emetica** (L.f.) Pers.		GAS (**emetica** = emetic)^*∗*^	emetic^*∗*^, anti-inflammatory, antineoplastic ^*∗*^, expectorant, to treat hemorrhoids ^*∗*^, diaphoretic, and vermifuge [151, 152]	Antimicrobial, antitumor, and antiproliferative activities [153]
		*Psychotria * **emetica** L.f.				

**Rutaceae** (4 species)	*Angostura trifoliata* (Willd.) T.S.Elias	*Cusparia * **febrifuga** Humb. ex DC.		MET (**febrifuga** = antipyretic)^*∗*^	Tonic, antidiarrhoeal, diaphoretic, sweating^*∗*^, and bronchitis [154]	ND
	*Conchocarpus toxicarius* (Spruce ex Engl.) Kallunki & Pirani ♦	*Cusparia * **toxicaria** Engl.		POI (**toxicaria** = toxic, containing a poisonous substance)	ND	ND
		*Galipea * **toxicaria** Spruce ex Engl.				
	*Esenbeckia febrifuga* (A.St.-Hil.) A.Juss. ex Mart. ♦	*Evodia * **febrifuga** A.St.-Hil.		MET (**febrifuga** = antipyretic)^*∗*^	Fevers^*∗*^, adenitis, dyspepsia, malaria^*∗*^, and constipation [155]	Antimalarial ^*∗*^ [156, 157]
		*Esenbeckia * **febrifuga** (A.St.-Hil.) A.Juss. ex Mart.				
	*Galipea jasminiflora* (A.St.-Hil.) Engl.♦	*Galipea * **febrifuga** (A.St.-Hil.) Baill.		MET (**febrifuga** = antipyretic)^*∗*^	Against venereal warts, astringent, intermittent fevers^*∗*^, dyspepsia, antidiarrhoeal, infections, malaria, and venereal warts [158, 159]	Treatment for intermittent fever^*∗*^ [86]
		*Ticorea * **febrifuga** A.St.-Hil.				

**Salicaceae** (1 species)	*Xylosma salzmannii* (Clos) Eichler ♦	*Xylosma salzmanni* Eichl.	**Quarenta- feridass/sessenta- feridas**	INF (**feridas** = wounds)^*∗*^	Astringent and dye [160]	ND

**Sapindaceae** (1 species)	*Matayba purgans* (Poepp.) Radlk. ♦	*Matayba * **purgans** (Poepp.) Radlk.		GAS (**purga** = purgative)	ND	ND

**Scrophulariaceae** (1 species)	*Capraria biflora* L.♦	*Capraria biflora* L.	**chá-bravo**	POI (**chá-bravo** = wild tea)	For kidneys, stomach pains, ulcers, hemorrhoids, diarrhoea, vomiting, pain, flu, fever, rheumatism [161, 162]	Antibacterial, antitumor, analgesic, and anti-inflammatory activity [163]

**Solanaceae** (1 species)	*Solanum stagnale* Moric. ♦	*Solanum stagnale* Moric.	**não-me-toque**	POI (**não-me-toque** = do not touch me)	ND	ND

**Tropaeolaceae** (1 species)	*Tropaeolum pentaphyllum* Lam. ♦	*Tropaeolum pentaphyllum* Lam.	**Chagas/chagueira**	INF (**chagas/chagueira** = Chagas disease)	Cold and diabetes [164]	Anticoagulant [165]

**Urticaceae** (2 species)	*Urera caracasana* (Jacq.) Gaudich. ex Griseb. ♦	*Urera caracasana* (Jacq.) Griseb.	**Urtiga-brava-de-fogo**	POI (**urtiga** = Nettle)	Relief from inflammation, diabetes, blood purification [166–168]	ND
	*Urtica urens* L.	**Urtica urens** L.		POI (**Urtica** = sting; *urens* = acrid, stinging, burning, to burn)^*∗*^	Allergy and atopic dermatitis^*∗*^ antidiabetic, diuretic, depurative, and antirheumatic [169]	Tonic, sedative, analgesic, chemoprotective and anti-inflammatory activity, phytodermatoses^*∗*^ [169, 170]

^*∗*^The 22 species whose “ethno” and pharmacological data are equivalent to plant lexicons. ♦ The 56 species native to Brazil. Bioprospecting categories: antidote (ANT), cancer (CAN), cardiovascular diseases (CAR), dermatologic disorders (DER), ophthalmic problems (EYE), gastrointestinal problems (GAS), gynecology (GYN), infections (INF), metabolic syndromes (MET), nervous system (NER), parasites (PAR), poisons (POI), respiratory complaints (RES), and urology (URO). ND: no data found. The possible medicinal and/or toxic property column follows the bioprospecting classification.

## Data Availability

The data used to support the findings of this study are included within the article.
